# A missing carbon sink from land to the ocean: Process and assessment

**DOI:** 10.1016/j.xinn.2026.101536

**Published:** 2026-08-11

**Authors:** Quanrui Chen, Fanglue Jiao

**Affiliations:** 1 Carbon Neutral Innovation Research Center and Fujian Key Laboratory of Marine Carbon Sequestration, Xiamen University, Xiamen 361005, China; 2 Global Ocean Negative Carbon Emissions Program Alliance, Xiamen, China; 3 Institute of Marine Science and Technology, Shandong University, Qingdao 266237, China

## A missing process in forest carbon-sink assessment

Forests are among the most visible and widely recognized terrestrial carbon sinks, and afforestation and reforestation are often regarded as feasible nature-based options for climate mitigation. Forest carbon sinks are usually assessed by measuring carbon retained within forest boundaries, including aboveground biomass, roots, litter, dead wood, and soil organic carbon. This plot-based view is essential for inventories, restoration assessments, and mitigation projects. Yet forests are open biogeochemical systems with a less visible dissolved carbon outlet: forest-derived dissolved organic carbon (DOC) that is leached from soil and transported by water.

The critical knowledge gap concerns the fate of exported dissolved carbon after it enters aquatic systems. Humified forest-soil organic matter is mobilized by runoff, groundwater flow, and erosion and transported through inland waters, estuaries, and shelves, where microbial, photochemical, mineral, and hydrological processes transform it. After partial degradation, part may enter refractory DOC (RDOC) reservoirs that persist over centennial-to-millennial timescales.^1^ This lateral DOC export and downstream RDOC formation are generally omitted from project-level forest carbon accounting, although they remain part of the broader land-ocean carbon cycle.

**Figure 1:**
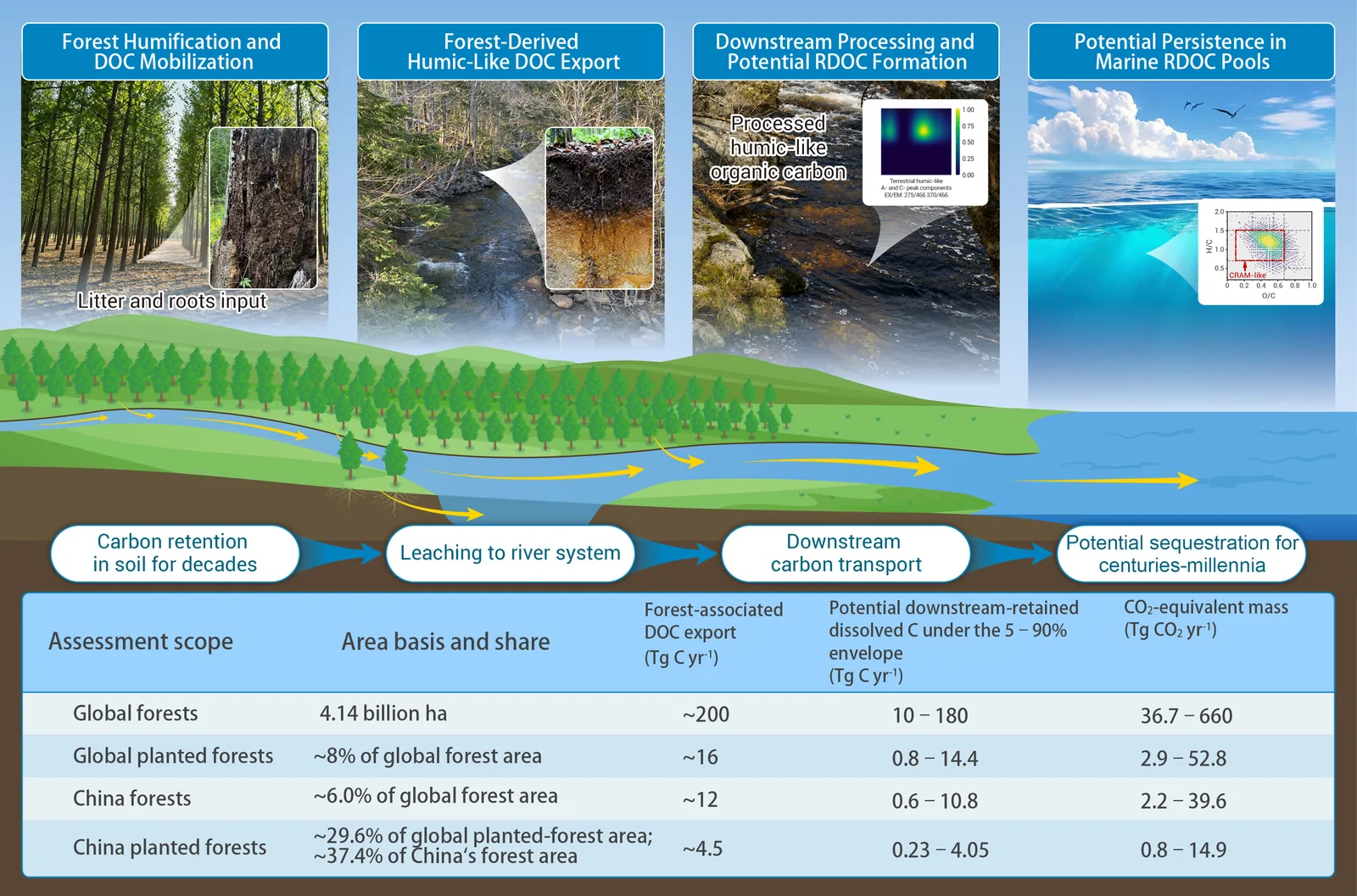
The land-ocean humic carbon pathway and potential downstream dissolved carbon preservation Forest-derived DOC is mobilized from soils, exported through inland waters, and progressively processed along the land-ocean continuum; only a fraction may ultimately contribute to marine RDOC. The bottom image presents area-scaled estimates of potentially retained forest-derived DOC for global forests, global planted forests, China’s forests, and China’s planted forests. Estimates were derived from global forest-stream DOC export,^2^ forest-area statistics,^3^^,^^4^ and illustrative retention scenarios of 5%–90%. This envelope is only a broad scenario range intended to encompass contrasting global environmental conditions, bracket the potential magnitude, and identify the observations needed to narrow the range in future assessments.

Terrestrial humification provides an intuitive starting point: dead wood, litter, roots, and humus-rich soils are visible and inventory-ready carbon pools, whereas the persistence of mobilized DOC depends on its transformation history and downstream processing. Brown or tea-colored waters draining forested catchments provide a visible expression of humic DOC mobilization, yet the transition from visible terrestrial carbon pools to less visible, long-lived dissolved reservoirs remains poorly represented in forest carbon-sink assessment (Figure 1).

We refer to this connection as the land-ocean humic carbon pathway. It captures a simple but overlooked possibility: forests, especially planted forests and shelterbelts, may alter production, leaching, and downstream transformation of humic-like DOC and RDOC precursors. Only the fraction that survives successive aquatic processing and persists in marine waters contributes to RDOC preservation. This pathway is most relevant where large-scale planting is hydrologically connected to river networks, deltas, continental shelves, and active marginal seas. The land-ocean continuum also transports particulate organic carbon, whose preservation is governed mainly by erosion, sediment transport, mineral association, and sedimentary burial.

The missing component in conventional forest carbon-sink assessment is the downstream fate of DOC exported beyond the forest boundary. Terrestrial DOC export and aquatic RDOC formation are established processes. By linking them within a forest source-to-sink framework, the land-ocean humic carbon pathway extends forest assessment beyond within-boundary stocks to the downstream fate and persistence of exported DOC. Its carbon-sink significance lies in the fraction that survives aquatic processing and persists as RDOC. Because forest-associated DOC and RDOC may contain both contemporary forest carbon and legacy soil organic carbon, attribution to recent afforestation, reforestation, or forest management requires an appropriate baseline that separates the incremental contribution from the pre-existing catchment signal.

Humic-like signals can support source attribution along this pathway through water color, chromophoric dissolved organic matter absorption, fluorescence components, spectroscopic indices, and molecular formulae resolved by high-resolution mass spectrometry.^1^ Attribution can be strengthened by sampling along river-estuary-shelf–open-ocean gradients and comparing molecular fingerprints among forest-draining waters, other terrestrial end members, and offshore marine waters. Molecular features enriched in forest-draining waters but absent or depleted in other end members can provide qualitative evidence of forest-associated transport and downstream transformation, although quantitative attribution requires further end-member constraints and mixing analysis.

## Scaling of downstream dissolved-carbon preservation

Quantitative assessment requires separating the forest-derived DOC source flux from the fraction retained downstream and the subset ultimately persisting as marine RDOC. Forest streams discharge approximately 200 Tg C year^−1^ as DOC.^2^ Because global observations remain insufficient to constrain a representative downstream-retention rate, we use 5%–90% solely as an illustrative global sensitivity envelope. This range applies to forest-stream DOC that has already undergone microbial transformation and hydrological selection during litter decomposition, soil processing, and leaching. Continued microbial, photochemical, and chemical processing may further alter it along the river-ocean continuum. The interval is neither an observed global mean nor a best estimate; it defines the potential magnitude space and the observations needed to narrow it.

Applying this illustrative envelope to the global source term gives 10–180 Tg C year^−1^ of potential downstream-retained dissolved carbon, equivalent by mass to 36.7–660 Tg CO_2_ year^−1^. Planted forests represent the managed subset of this broader pathway. Because planted forests occupy approximately 8% of global forest area,^3^ first-order area scaling gives approximately 16 Tg C year^−1^ of DOC export and a potential downstream-retained range of 0.8–14.4 Tg C year^−1^, equivalent to 2.9–52.8 Tg CO_2_ year^−1^. These values are scenarios rather than realized global sink estimates.

China provides a globally significant managed case because it contains approximately 29.6% of the world’s planted-forest area. Official statistics report about 247 million ha of forest and 92.4087 million ha of retained planted-forest area,^4^ equivalent to 6.0% of global forest area, while planted forests account for 37.4% of China’s forest area. Area scaling gives about 12 Tg C year^−1^ of forest-associated DOC export and a potential downstream-retained range of 0.6–10.8 Tg C year^−1^ (2.2–39.6 Tg CO_2_ year^−1^). China’s planted forests contribute about 4.5 Tg C year^−1^ of DOC export and a retained range of 0.23–4.05 Tg C year^−1^ (0.8–14.9 Tg CO_2_ year^−1^). This scale offers an exceptional opportunity to test how afforestation, management, and hydrological connectivity regulate DOC export and potential RDOC preservation.

Area-based scaling cannot replace regional process models, but it provides a transparent global reference for comparing potential magnitudes. Forest-derived DOC transport may occur over days to years, whereas the fraction that survives marine processing and enters persistent RDOC pools can remain for centuries to millennia.^1^ This transport interval creates a time lag between DOC export and RDOC preservation, and the final preserved fraction depends on cumulative processing losses along the pathway. Compared with many tree-biomass carbon pools, preserved RDOC may therefore represent a smaller annual flux but a more persistent form of carbon storage.

## Toward RDOC-centered source-to-sink assessment

RDOC-centered source-to-sink assessment asks how forest humification changes DOC leaching, how inland and coastal processing transforms that signal, and where part of it becomes durably preserved in RDOC reservoirs. A forest-derived RDOC-forming pathway is most relevant when humic-like DOC or RDOC-precursor signals can be attributed to forest sources, traced through connected waters, and linked to longer-lived dissolved reservoirs. The Triple REAL filters provide a useful screening logic by asking whether such a pathway is technically reproducible, environmentally acceptable, action oriented, and legally accountable,^5^ but the central assessment object here remains DOC leaching, RDOC formation, residence time, and preservation.

China’s planted forests offer a practical managed case for this assessment. Their stand structure, species composition, disturbance history, and management intensity may regulate litter and root inputs, soil humification, hydrological connectivity, and DOC leaching. Dense vegetation can also reduce erosion and sediment export, helping separate the dissolved pathway from sediment-driven particulate organic carbon transport. These effects remain testable hypotheses requiring paired observations, source attribution, and downstream persistence.

Forest carbon retained *in situ*, DOC exported beyond the forest boundary, and RDOC preserved downstream are linked stages of one carbon trajectory and should not be summed as independent sinks. Any accounting claim should therefore be limited to the net preserved fraction and specify its source area, baseline, flow path, receiving reservoir, time lag, residence time, uncertainty range, and assigned responsibility. This separation reduces double counting and supports a stepwise application of the pathway: first as scientific recognition in carbon-cycle research, then as a component of regional carbon-budget and ecosystem-service assessment, and eventually as a basis for methodological development where attribution, additionality, persistence, uncertainty, and verification can be demonstrated.

This illustrative sensitivity assessment opens a rarely quantified assessment space. Planted forests and shelterbelts may influence DOC leaching, RDOC-precursor export, and long-term downstream preservation alongside terrestrial biomass and soil carbon storage. Recognizing the land-ocean humic carbon pathway therefore extends forest carbon assessment toward a source-to-sink evaluation of carbon fate and persistence across terrestrial, riverine, and ocean reservoirs.

## Funding and acknowledgments

This work was supported by the 10.13039/501100001809National Natural Science Foundation of China (grant no. 42188102) and the Global Ocean Negative Carbon Emissions (ONCE) program.

## Declaration of interests

The authors declare no conflicts of interest.
